# Effectiveness Between Daily and After-Each-Case Room Disinfection of the Endoscopy Unit

**DOI:** 10.3389/fpubh.2021.700041

**Published:** 2021-10-05

**Authors:** Bo Jin, Yue Hu, Liang Huang, Xiaoyun Cheng, Jin Zhao, Xuejing Yang, Xiling Sun, Tieer Gan, Bin Lu

**Affiliations:** ^1^Department of Endoscopy Center, the First Affiliated Hospital of Zhejiang Chinese Medical University, Hangzhou, China; ^2^Department of Gastroenterology, First Affiliated Hospital of Zhejiang Chinese Medical University, Hangzhou, China; ^3^Key Laboratory of Digestive Pathophysiology of Zhejiang Province, First Affiliated Hospital of Zhejiang Chinese Medical University, Hangzhou, China; ^4^Department of Clinical Laboratory, First Affiliated Hospital of Zhejiang Chinese Medical University, Hangzhou, China; ^5^Department of Hospital-Acquired Infection Control, First Affiliated Hospital of Zhejiang Chinese Medical University, Hangzhou, China

**Keywords:** gastroscopy, bacteria, endoscopy unit, air disinfection, fungi, chemical disinfection

## Abstract

**Background:** To evaluate the effectiveness between daily and after-each-case room disinfection in the endoscopy unit.

**Methods:** This study was conducted in an endoscopy unit of the First Affiliation of Zhejiang Chinese Medical University. We cultured samples from the surface of endoscopy unit items, including operation unit air, isolation gown of an endoscopist, control panel buttons, workstation mouse, and the bed head of the patient. All the samples were divided into daily and after-each-case room disinfection groups. In addition, each group was subdivided into sedation and nonsedation gastroscopy with and without ventilation room groups.

**Results:** The qualified rate of bed head samples of the patient were lower in the daily room disinfection group (76.67%) compared with the after-each-case group (100%). The isolation gown, mouse at the workstation, and the bed head of the patient demonstrated the lowest bacterial and fungal load in the after-each-case room disinfection group compared with the daily room disinfection group (*p* < 0.05). In the subgroup analysis, a higher microbial load was observed for the isolation gown of the endoscopist used during nonsedation gastroscopy in an unventilated room under the after-each-case room disinfection pattern (*p* < 0.05); a higher microbial load was observed for the control panel buttons used during nonsedation gastroscopy under the after-each-case room disinfection pattern (*p* < 0.05).

**Conclusions:** For risk-free or low-risk patients, daily room disinfection provides the basic health requirements of the endoscopy procedure. However, it is better to adopt the after-each-case room disinfection for the isolation gown of the endoscopist and bed head of the patient. For the patients with high risk, the after-each-case room disinfection is more suitable for every endoscopy unit (www.ClinicalTrials.gov, NCT04399005).

## Introduction

In recent decades, the introduction of the flexible endoscope has become an important tool for diagnosing, monitoring, and managing gastrointestinal disorders. However, patients or healthcare personnel could be infected with various harmful pathogens through the contaminated endoscope and/or endoscopy unit environment as an invasive procedure. Thus, standardized and efficient disinfection was essential to minimize the risk of infection from microbial contamination.

Many clinical guidelines for quality control were published; most of which focused primarily on the standardized decontamination of endoscopes, which was of less attention to the environment at the endoscopy center (1–3). With respect to the current guidelines, it was recommended that the procedural space of the endoscopy unit should be cleaned and disinfected at the end of the day (4, 5). For the patients with, or suspected of having, *Clostridium difficile*, airborne, viral, or other highly contagious diseases, mentioned in the guidelines, rigorous cleaning of the endoscopy unit for environmental disinfection after an endoscopic procedure was required (5). Given that it was still in the period of the COVID-19 epidemic, many national and international associations, including the American College of Gastroenterology (6), the British Society of Gastroenterology (7), the Asian Pacific Society for Digestive Endoscopy (APSDE) (8), and the Chinese Society of Digestive Endoscopy, have proposed guidelines for the endoscopic centers in terms of management and preventive measures for special infectious diseases (9), emphasizing complete disinfection of the endoscopy room and equipment after-each-case room examination.

Unlike conventional daily room disinfection (10), the after-each-case room disinfection would ensure the safety of endoscopy personnel and patients, which would limit the efficiency of inspection and increase the time and cost, especially in the areas with a shortage of supplies and/or a large number of patients. In addition, endoscopy centers are often facing the challenge of maintaining an adequate balance between the growing demand for gastrointestinal endoscopy and the strict disinfection guidelines of the operating unit. However, there is still a lack of knowledge on comparing these two methods of disinfection followed at the endoscopy center.

Our objective was to evaluate the effectiveness of daily and after-each-case room disinfection in the endoscopy unit to determine the right one to ensure daily clinical needs.

## Methods

### Sample

**Figure 1:**
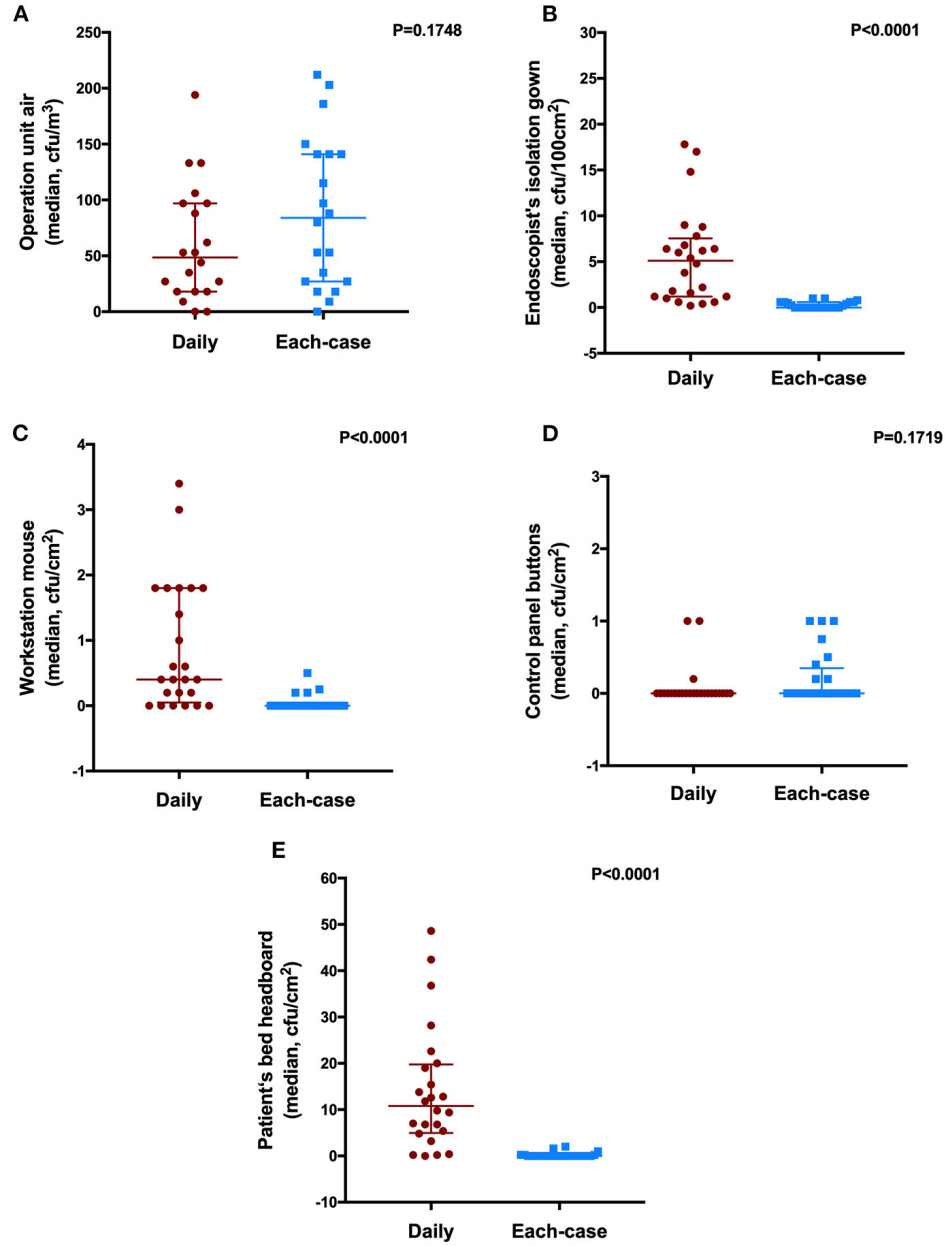
Sampling diagram in the endoscopy unit. **(A)** operation unit air; **(B)** isolation gown of the endoscopist; **(C)** bed head of the patient; **(D)** control panel buttons; and **(E)** workstation mouse.

This was a single-center, prospective, pilot study conducted in an endoscopy unit of the First Affiliation of Zhejiang Chinese Medical University. The study was approved by the institutional review board (2020-KL-024-01) and registered on www.ClinicalTrials.gov (NCT04399005). The following elements of the endoscopy unit were examined: air in the operation unit, the isolation gown of the endoscopist, control panel buttons, mouse at the workstation, and bed head of the patient (Figure 1). Information of the patient was not collected during this study.

Gastroscopic procedures were performed on the weekdays between 8:00 am and 5:00 pm in three endoscopy rooms. The samples were collected from May 25, 2020, to June 5, 2020. All the patients were excluded from high-risk personal through epidemiological history, symptoms, body temperature, COVID-19 virus nucleic acid test, and CT scan of the chest. The cases included inpatient and outpatient gastroscopic procedures performed in the sedated or nonsedated patients. Endoscopists performed all the procedures and anesthesiologists provided deep sedation. During endoscopy, healthcare personnel must wear proper personal protective equipment, including N95 or surgical masks, isolation gowns, gloves, and eye protection with goggles or a facial shield.

Samples collected were as follows: for the air sample of the operation unit, a six-stage sieve percussion air sampler (airflow rate: 28.3 L/min; sampling time: 4 min) was used; for the workstation mouse, sterile cotton was used to wipe the surface of the mouse; and for isolation gown of the endoscopist, the control panel buttons, and the bed head of the patient, a contact plate was used.

### Study Design

The study samples were divided into the following two groups: daily and after-each-case room disinfection groups. Each group was again subdivided into sedation and nonsedation gastroscopy with or without ventilation operation room. Room disinfection was defined as the discarding of the isolation gown of the endoscopist, cleaning, and disinfection of the surface of the facility (including control panel buttons, mouse at the workstation, the cover of a bed of the patient, etc.) and the air in the endoscopy room after the examination. Specifically, sanitizing wipes containing quaternary ammonium salt, alcohol, and interfacial activator were used to wipe the surfaces and the air disinfection machine was continuously turned on for 30 min (9). Daily room disinfection was defined as disinfection after completing eight nonsedation gastroscopies or four sedation gastroscopies; the after-each-case room disinfection was defined as disinfection performed after completing the examination of each patient. In our center, the bed of the patient had a cover, which would be covered with a new disposable upper drape for each patient. After performing gastroscopy for a patient, this disposable upper drape would be discarded; then, endoscopy personnel would change gloves and perform a routine hand hygiene protocol (using a fast hand disinfectant containing ethanol) before attending the next patient (11). After the final gastroscopy examination, the bed cover was thrown away.

### Review of Microorganisms

Nutrient petri plates inoculated with individual samples were incubated at 35°C for 48 h. The colony count was then performed and equated to colony-forming units (CFUs) per plate area. Microbial detection was interpreted by two medical laboratory pathologists. The details of microbial detection are provided in Supplementary File 1.

According to our study protocol, unacceptable levels of bioburden were >10 CFU/cm^2^ for mouse samples at the workstation, control panel buttons, and bed head of the patient; >200 CFU/100 cm^2^ for samples of isolation gown of the endoscopist; and >500 CFU/m^3^ for air samples from the operating unit (12).

### Data Analysis

All the data were statistically analyzed using SPSS Statistics version 22.0 (IBM Corporation, Somers, New York, USA) by a statistician from the Clinical Evaluation and Analysis Center of the First Affiliated Hospital of Zhejiang Chinese Medical University. Continuous variables with a nonnormal distribution or ordinal variables are expressed as median (the lower four quantiles, the upper four quantiles). Categorized variables are summarized as counts and proportions. Continuous categorical variables were compared using the Wilcoxon signed-rank test (abnormal distribution), while categorical variables were compared using the Fisher's exact test or the chi-squared test. All *p*-values were two-sided, with a CI of 95%. *p* < 0.05 was considered as statistically significant. All the authors had access to the study data and reviewed and approved the final manuscript.

## Results

### Overall Eligibility

During the study period, 240 gastroscopies were performed in three endoscopy rooms and 1,020 samples were obtained. All the positive culture results from ambient air (<500 CFU/m^3^), isolation gown of the endoscopist (<200 CFU/100 cm^2^), and control panel buttons (<10 CFU/cm^2^) were within the qualified values. Only once, a sample of the mouse at the workstation from the daily room disinfection group demonstrated >10 CFU/cm^2^. It should be noted that the qualified rate was not significantly different in the groups. Interestingly, regardless of whether the room was ventilated or not and whether gastroscopy was performed with or without sedation, the qualified rate of bed head samples of the patient was lower in the daily room disinfection group [76.67% (92/120) of the samples] compared with the after-each-case room disinfection group [100% (120/120) of samples] (Table 1).

**Table 1:** The qualified rate of cultured samples from the surface of endoscopy unit items between the daily and the after-each-case room disinfection groups.

**Items**	**Daily (*n*, %)**	**Each-case (*n*, %)**	***P*-value**
Operation unit air	20 (100)	20 (100)	-
Endoscopist's gown	120 (100)	120 (100)	-
Workstation mouse	119 (99.17)	120 (100)	0.3163
Control panel buttons	120 (100)	120 (100)	-
Patient bed head	92 (76.67)	120 (100)	<0.0001

*N, number of qualified tests*.

*%, Qualified rate*.

### Daily vs. After-Each-Case Room Disinfection

**Figure 2:**
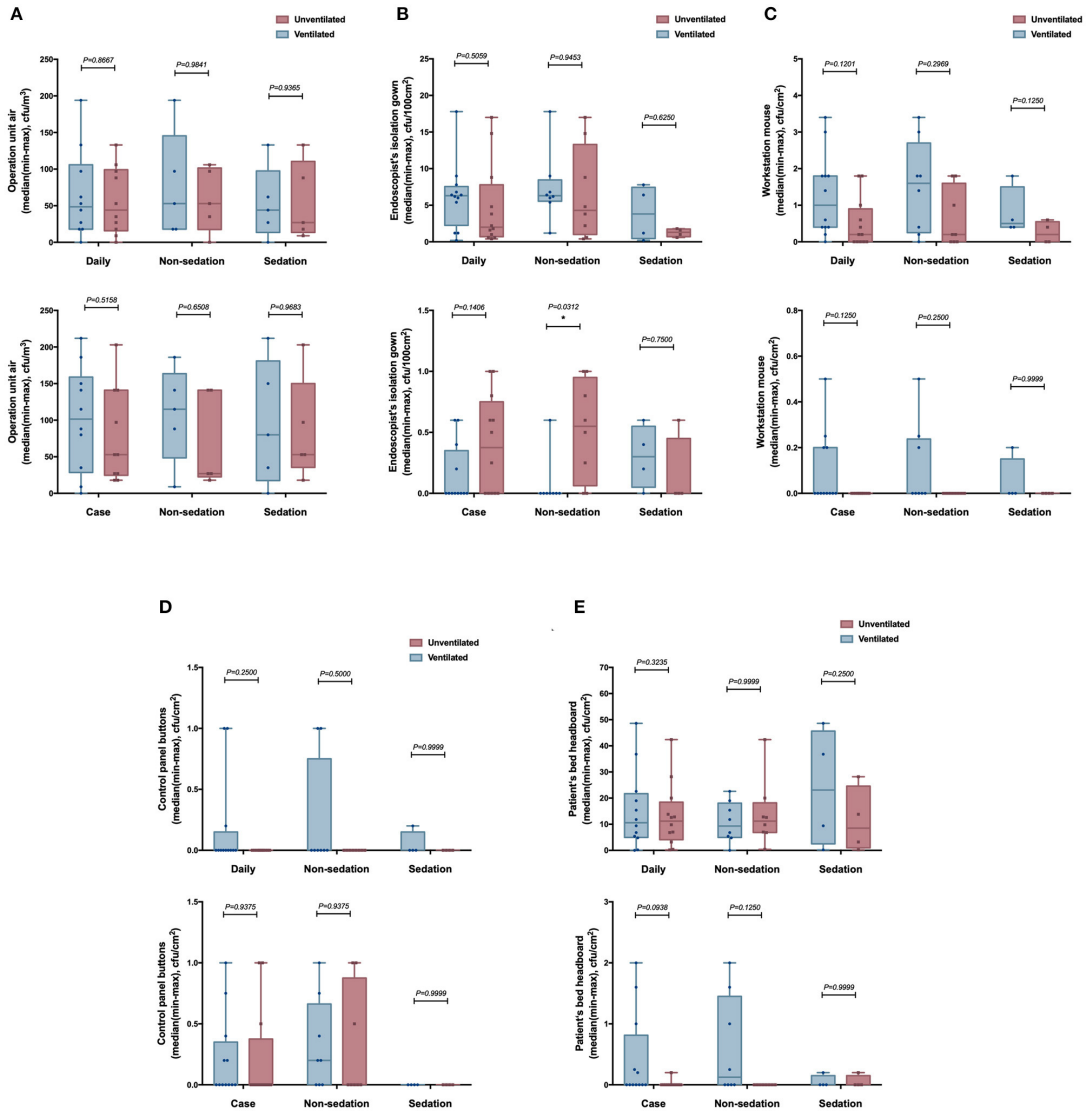
The number of culture samples in the level of colony-forming units (CFUs) for the surface of the endoscopy unit between the daily room disinfection and the after-each-case room disinfection. **(A)** operation unit air; **(B)** isolation gown of the endoscopist; **(C)** workstation mouse; **(D)** control panel buttons; and **(E)** bed head of the patient.

A comparison of the samples was performed between the daily and the after-each-case room disinfection groups. As shown in Figure 2, we found that the surfaces of the isolation gown, mouse workstation, and bed-head demonstrated the lowest bacterial and fungal load in the after-each-case room disinfection group compared with the daily disinfection group (*p* < 0.05). However, we found no difference in microbial load for the air of the operation unit and the control panel between the disinfection groups (*p* > 0.05).

### Subgroup Analysis (Ventilated vs. Unventilated Room)

**Figure 3:**
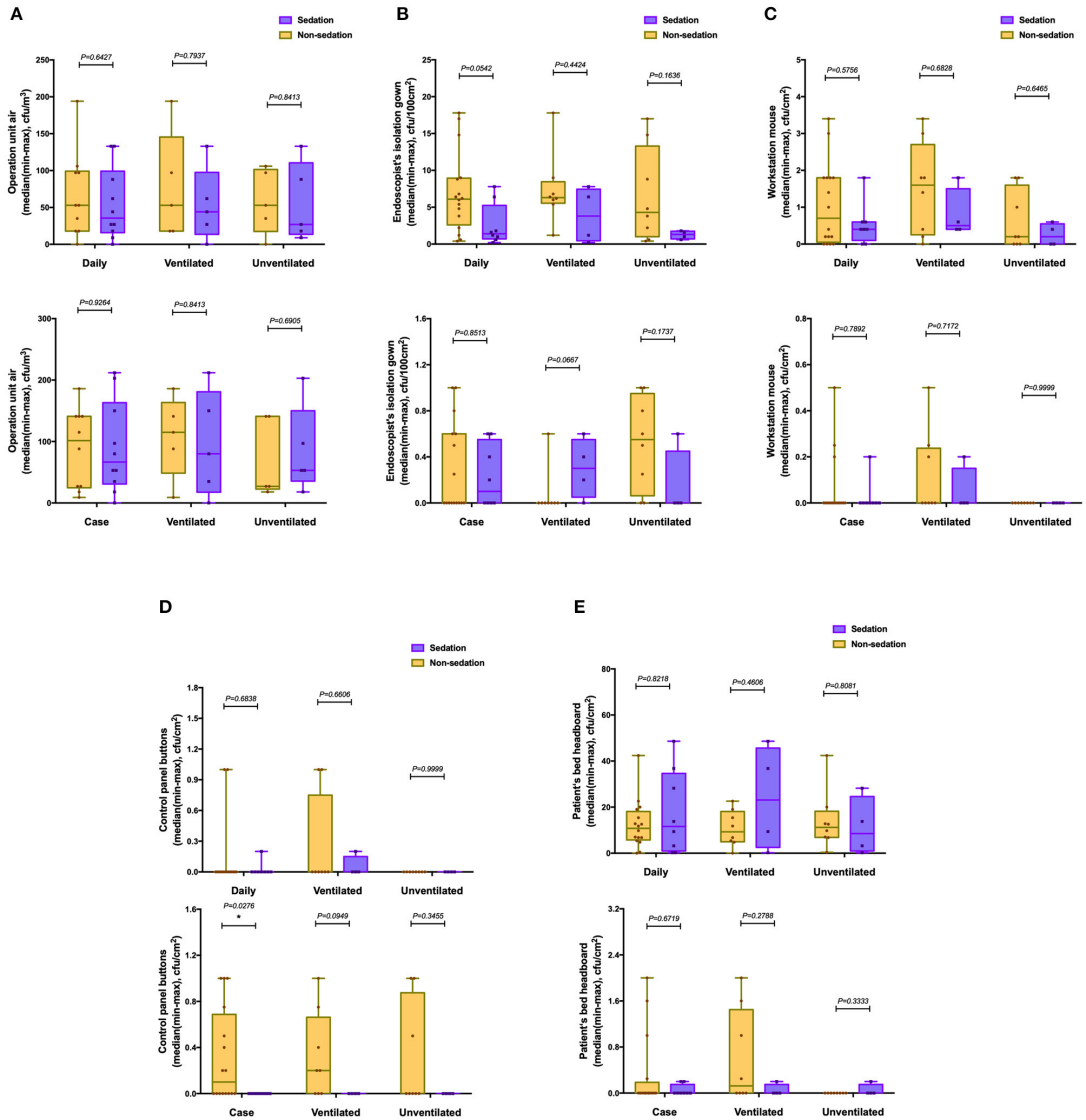
The number of culture samples in the level of CFUs for the surface of the endoscopy unit between ventilated and unventilated rooms. **(A)** operation unit air; **(B)** isolation gown of the endoscopist; **(C)** workstation mouse; **(D)** control panel buttons; and **(E)** bed head of the patient. *p < 0.05.

For the subgroup analysis with or without ventilation (Figure 3), we found that the microbial load on the surface of the isolation gown of the endoscopist used during nonsedation gastroscopy in the unventilated room was higher than that in the ventilated room in the after-each-case room disinfection group (*p* < 0.05, Figure 3B). Except for this difference, we found no other statistically significant differences between other pairs of the groups (*p* > 0.05).

### Subgroup Analysis (Sedation vs. Nonsedation Gastroscopy)

**Figure 4:**
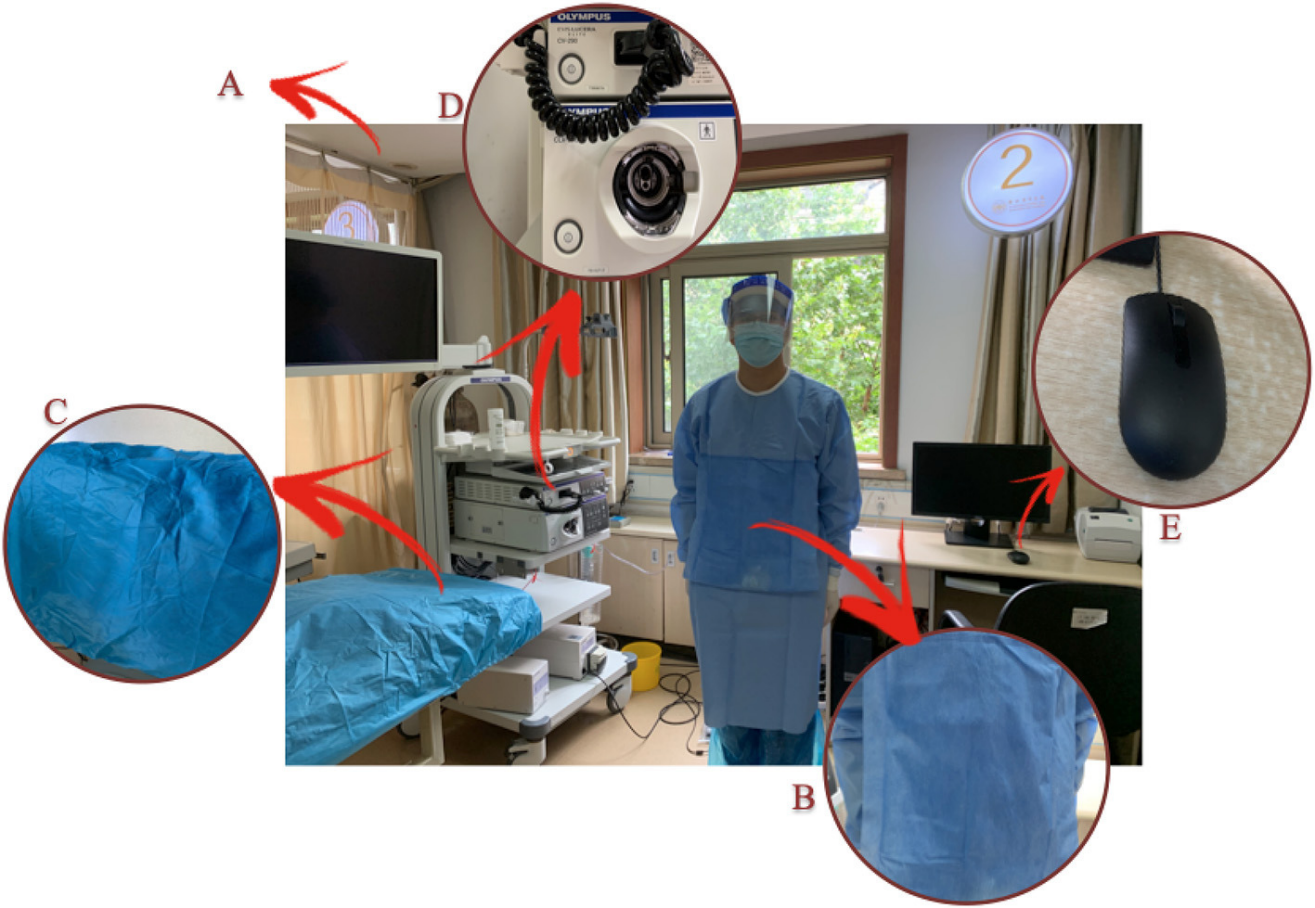
The number of culture samples in the level of CFUs for the surface of the endoscopy unit between sedation and nonsedation gastroscopy. **(A)** operation unit air; **(B)** isolation gown of the endoscopist; **(C)** workstation mouse; **(D)** control panel buttons; and **(E)** bed head of the patient.

For the subgroup analysis of sedation and nonsedation gastroscopy (Figure 4), the surface of control panel buttons used during nonsedation gastroscopy had a higher microbial load than those used during sedation in the after-each-case room disinfection group (*p* < 0.05, Figure 4D). Except for this difference, we found no other statistically significant differences between other pairs of the groups (*p* > 0.05).

## Discussion

Gastroscopy is an invasive and operator-dependent method that poses a great risk of virus and/or bacterial transmission. Thus, establishing and maintaining general cleanliness and disinfection control standards within an endoscopy unit is essential to create a high-quality safety environment for the patients and personnel. High-level disinfection of the endoscopy can reduce or kill potential pathogenic microorganisms, preventing the transmission of pathogenic microorganisms between the patients (5, 10). However, data to confirm the effectiveness of the environmental disinfection of the endoscopy unit are limited. To the best of our knowledge, this is the first prospective clinical trial to evaluate the difference between different disinfection patterns, comprising daily and the after-each-case room disinfection in the endoscopy unit.

Several viruses and other pathogens have an airborne route of transmission, placing the patients and healthcare personnel exposed to ambient air in the same area at an increased risk of contracting the disease. Therefore, attention must be paid to monitoring air quality in the endoscopy unit (5, 13). In this study, we found no significant differences between any of the culture results of operation unit air, including in the prespecified subgroup analysis. Furthermore, all the microbial loads in the culture results were <500 CFU/m^3^ (12), in compliance with national disinfection standards of the endoscopy unit. Our findings revealed that daily disinfection could meet operation safety requirements, regardless of room ventilation and patient sedation status. During the COVID-19 or other infectious pandemic disease, it is appropriate to perform gastroscopy and other endoscopic operations in a ventilated room and daily disinfection appears adequate to maintain air quality. However, it must be emphasized that the unit must be cleaned and disinfected immediately after the gastroscopy of the high-risk patients.

In terms of the health and safety of endoscopy personnel, global guidelines recommend that the treating personnel should wear appropriate protective clothing with face masks or eyeglasses (5, 7–9). In our study, the isolation gown was tested as it has closer contact with the patient with a relatively high possibility of microbial contamination. The results indicated that the disinfection of the gown was more effective in the after-each-case room disinfection group compared with the daily disinfection group, but the microbial load in all cultures of the gown samples was within the reference range. Meanwhile, we found that it might be beneficial to perform nonsedation gastroscopy in a ventilated room under the after-each-case room disinfection conditions. This, in combination with our result, might be acceptable that the gown of the endoscopist could be disinfected daily only if the location has deficit medical supplies and the high-risk patients were excluded; else, it required replacement for each case.

The head of the patient bed is also a place that is easily contaminated and often overlooked in addition to ambient air and isolation gown. We found that the bacterial load of the bed head of the patient was higher in the daily room disinfection group compared with the after-each-case room disinfection group and with the lowest qualified rate. Although the daily environmental disinfection of the endoscopy unit is based on the guidelines, it seems not to work effectively when performed on the bed head of the patient. Therefore, we recommend that the bed head can be cleaned using the after-each-case room disinfection while performing routine cleaning and disinfection of the endoscopy unit.

The culture results of the control panel buttons and mouse at the workstation met the required qualifying standards, indicating that daily room disinfection could satisfy the health requirements of these two places in the endoscopy unit. Specifically, the after-each-case room disinfection was better than daily room disinfection to reduce microbial accumulation on the mouse surface, but we found no difference in the two disinfection procedures on control panel buttons. This finding could be attributed to hand hygiene; in our center, experienced assistant nurses operate the control panel buttons, but both the operators and the nurses operate with the mouse. According to published studies and guidelines (5, 14), good hand hygiene minimizes the risk of transmission of an infection to a great extent. Thus, overall, our results also reflect the importance of hand hygiene that should be emphasized for the safety of the patient and quality assurance in gastrointestinal endoscopic procedures.

With respect to the subgroups, several studies have reported that patients receiving gastroscopy with sedation have lesser throat response (15, 16), which might reduce the contamination of aerosol and other airborne droplets. Meanwhile, it is also well recognized that increasing air circulation reduces microbial colonization. However, our results did not demonstrate significant differences between these two subgroups.

This study has certain limitations that warrant further examination. First, this was a single-center prospective study with a relatively small sample size. Second, we only cultured for bacteria and fungi and not for viruses that could considerably limit the assessment. Although the transmission of viruses is not the same as that of bacteria and fungi, the detection and cultivation can reflect a certain degree of the virus. Third, not all bacteria are amenable to culture using the medium employed in this study leading to the possible omission of some pathogenic microorganisms. Future prospective multicenter studies are required to validate the findings of this study.

In summary, considering that there is still a spread of COVID-19 worldwide, endoscopy centers are recommended for infectious risk stratification of the patients before endoscopy examination. We recommend the after-each-case room disinfection for isolation gown of the endoscopist and bed head of the patient and the daily room disinfection for ambient air for the risk-free or low-risk patients, workstation mouse, and control panel buttons. However, for the high-risk patients, we highly recommend the after-each-case room disinfection for each endoscopy unit.

## Data Availability Statement

The raw data supporting the conclusions of this article will be made available by the authors, without undue reservation.

## Ethics Statement

The institutional review board approved the study protocol and informed consent form (the First Affiliated Hospital, Zhejiang Chinese Medical University; IRB number 2020-KL-024-01). The patients/participants provided their written informed consent to participate in this study.

## Author Contributions

BL designed and supervised the study including all data collection and analysis. BJ and YH performed most of the investigations, including data collection and analysis, and drafted the manuscript. LH, XC, JZ, and TG assisted with the data collection. XY and XS conducted a review of microorganisms. All the authors have read and approved the manuscript.

## Funding

This study was supported by the Natural Science Foundation of Zhejiang Province (No. LQ21H030001) and the Zhejiang Administration of Traditional Chinese Medicine (Grant No. 2020ZA051).

## Conflict of Interest

The authors declare that the research was conducted in the absence of any commercial or financial relationships that could be construed as a potential conflict of interest.

## Publisher's Note

All claims expressed in this article are solely those of the authors and do not necessarily represent those of their affiliated organizations, or those of the publisher, the editors and the reviewers. Any product that may be evaluated in this article, or claim that may be made by its manufacturer, is not guaranteed or endorsed by the publisher.
