# Supplementary material for: Effectiveness Between Daily and After-Each-Case Room Disinfection of the Endoscopy Unit
Source: Front Public Health. 2021 Oct 5;9:700041. doi: 10.3389/fpubh.2021.700041 (PMC8523938; doi:10.3389/fpubh.2021.700041)
Supplement: Supplementary file 1 [file Data_Sheet_1.DOCX]

**Microbial Detection After Sampling of the Endoscopy Unit Items**

**Methods**

**1. Materials**

**1.1 Reagents and instruments**

Eluent test tubes (9 mL/piece) containing the corresponding neutralizing agent (0.3% Tween-80 and 0.3% Lecithin) (Hangzhou Binhe, lot number: 200326), 9-cm nutrient agar plates (Wenzhou Kangtai, lot number: TC2303P), contact plates (manufacturer: Jiangmen Kailin, lot number: 200324), and a biochemical incubator.

**1.2 Quality Control**

ATCC25922 *Escherichia coli*, ATCC25923 *Staphylococcus aureus*, an unsampled blank nutrient agar plate, and an unsampled blank contact plate acted as negative controls.

**2. Detection method**

**2.1 Detection of air microorganisms**

The 9-cm nutrient agar plates were inoculated and then inverted and cultured at 35 °C for 48 h. Next, the number of colonies in each agar plate was counted.

**Operation unit air**: CFU/m^3^ = CFU per dish*6 dishes*1000/(28.3 L/min × 4 min)

**2.2 Detection of surface microorganisms**

**2.2.1 Microbiological examination of the headboard of patient’s bed**

After contacting the plates for sampling, they were culture at 35 °C for 48 h. Next, the colony numbers in each plate were counted. The number of colonies on the object surface = the number of colonies in the Petri dish/plate area.

**2.2.2 Microbiological examination of control panel buttons**

Contact plates with a microbial detection surface area of 25 cm^2^ containing the corresponding neutralizer was incubated at 35 °C for 48 h. Next, the number of bacterial colonies per dish was counted. The number of colonies on the object surface = the number of colonies in the Petri dish/plate area.

**2.2.3 Microbial examination of the workstation mouse**

After sampling, sterile cotton which was used to wipe the surface of the mouse was shaken in 9 mL/piece of the eluent containing the corresponding neutralizing agent, 200 mL/piece of the eluent was isolated and inoculated on a 9-cm nutrient agar plate, incubated at 35 °C for 48 h, and the number of colonies in a Petri dish was counted.

**2.2.4 Microbiological examination of the endoscopist’s isolation gown (abdominal area)**

After sampling, the contact plate with a surface area of 25 cm^2^ containing the corresponding neutralizer was cultured at 35 °C for 48 h, and the number of colonies per petridish was counted. The number of colonies on the object surface = the number of colonies in Petri dish/plate area.
